# Recombinant AAV Vectors for Enhanced Expression of Authentic IgG

**DOI:** 10.1371/journal.pone.0158009

**Published:** 2016-06-22

**Authors:** Sebastian P. Fuchs, José M. Martinez-Navio, Guangping Gao, Ronald C. Desrosiers

**Affiliations:** 1 Department of Pathology, Miller School of Medicine, University of Miami, Miami, Florida, United States of America; 2 Institut für Klinische und Molekulare Virologie, Friedrich-Alexander-Universität Erlangen-Nürnberg, Erlangen, Germany; 3 Gene Therapy Center, University of Massachusetts Medical School, Worcester, Massachusetts, United States of America; National Institute of Dental and Craniofacial Research, UNITED STATES

## Abstract

Adeno-associated virus (AAV) has become a vector of choice for the treatment of a variety of genetic diseases that require safe and long-term delivery of a missing protein. Muscle-directed gene transfer for delivery of protective antibodies against AIDS viruses and other pathogens has been used experimentally in mice and monkeys. Here we examined a number of variations to AAV vector design for the ability to produce authentic immunoglobulin G (IgG) molecules. Expression of rhesus IgG from a single single-stranded AAV (ssAAV) vector (one vector approach) was compared to expression from two self-complementary AAV (scAAV) vectors, one for heavy chain and one for light chain (two vector approach). Both the one vector and the two vector approaches yielded considerable levels of expressed full-length IgG. A number of modifications to the ssAAV expression system were then examined for their ability to increase the efficiency of IgG expression. Inclusion of a furin cleavage sequence with a linker peptide just upstream of the 2A self-cleaving sequence from foot-and-mouth disease virus (F2A) increased IgG expression approximately 2 fold. Inclusion of these sequences also helped to ensure a proper sequence at the C-terminal end of the heavy chain. Inclusion of the post-transcriptional regulatory element from woodchuck hepatitis virus (WPRE) further increased IgG expression 1.5–2.0 fold. IgG1 versions of the two rhesus IgGs that were examined consistently expressed better than the IgG2 forms. In contrast to what has been reported for AAV2-mediated expression of other proteins, introduction of capsid mutations Y445F and Y731F did not increase ssAAV1-mediated expression of IgG as determined by transduction experiments in cell culture. Our findings provide a rational basis for AAV vector design for expression of authentic IgG.

## Introduction

Gene therapy is a relatively modern research field that developed shortly after mapping of the simian virus 40 (SV40) genome in the early 1970s [1,2]. It soon became apparent that viral vectors could be used to transfer genetic material to humans with the intention of correcting hereditary disorders [3]. One of the first clinical trials utilized a retroviral vector to deliver adenosine deaminase (ADA) to individuals lacking this enzymatic activity [4]. Initial optimism was soon tempered by the occurrence of oncogenic transformation in some treated individuals as a consequence of retroviral insertional mutagenesis [5].

Risks and limitations are to be expected with any viral vector system. Notwithstanding, vector-based gene delivery using adeno-associated virus (AAV) has evolved to become a relatively safe and effective technology. The safety and the successful application of recombinant AAV (rAAV) vectors have been demonstrated in numerous studies [1,6–12]. A rAAV vector for the treatment of lipoprotein lipase deficiency (LPLD) is the first gene therapy product to achieve regulatory approval by a governmental health institute [13–15]. Several groups, including our own, are looking to extend the utility of AAV vectors by using them to deliver antibodies (Abs) and antibody-like molecules for the prevention and treatment of AIDS virus infection. Protective effects against simian immunodeficiency virus (SIV) in monkeys [16,17], simian-human immunodeficiency virus (SHIV) in monkeys [18,19] and human immunodeficiency virus (HIV) in humanized mice [20] have already been reported.

While the pioneering study of Johnson et al. [16] utilized self-complementary AAV (scAAV) to deliver the shorter antibody-like molecules in the form of single-chain fragment variable immunoadhesins (scFvi), subsequent studies have delivered authentic immunoglobulin G (IgG) molecules [17,19–21]. The genetic material encoding the immunoadhesins 4L6 and 5L7 used previously [16] was small enough to be accommodated by scAAV vector, a rAAV variant that encapsidates double-stranded DNA [22]. While scAAV has been shown to achieve higher rates of transgene expression than single-stranded AAV (ssAAV) [23], it cannot package the genetic information of both heavy and light chain sequences of a full-length IgG [24]. Furthermore, immunoadhesins are artificially composed molecules that do not reflect the natural structure of authentic immunoglobulins such as IgG. Thus, use of immunoadhesins suffers from the potential generation of artificial epitopes that could elicit immune responses [16]. It has also been reported that full-length IgG molecules may sometimes have higher potencies with regard to virus neutralization than their immunoadhesin counterparts [25].

As the field moves forward, it will be important to fully investigate variations in rAAV vector design so that the efficiency of antibody delivery can be optimized. Our work aims at constructing efficient IgG expression cassettes based on the previously reported immunoadhesin sequences 4L6 and 5L7 [16]. For this purpose we compared two strategies for the expression of rhesus IgG: from a single ssAAV vector (one vector approach) and from two scAAV vectors (two vector approach). Furthermore, we have examined a number of modifications to the ssAAV vector design and to the AAV1 capsid for their ability to improve the efficiency of IgG production. Sequence modifications in the region of protease cleavage between heavy and light chains and addition of a WPRE element were found to enhance expression, while Y-to-F mutations in the AAV1 capsid did not.

## Results

### Expression of full-length IgGs from rAAV vectors

**Fig 1:**
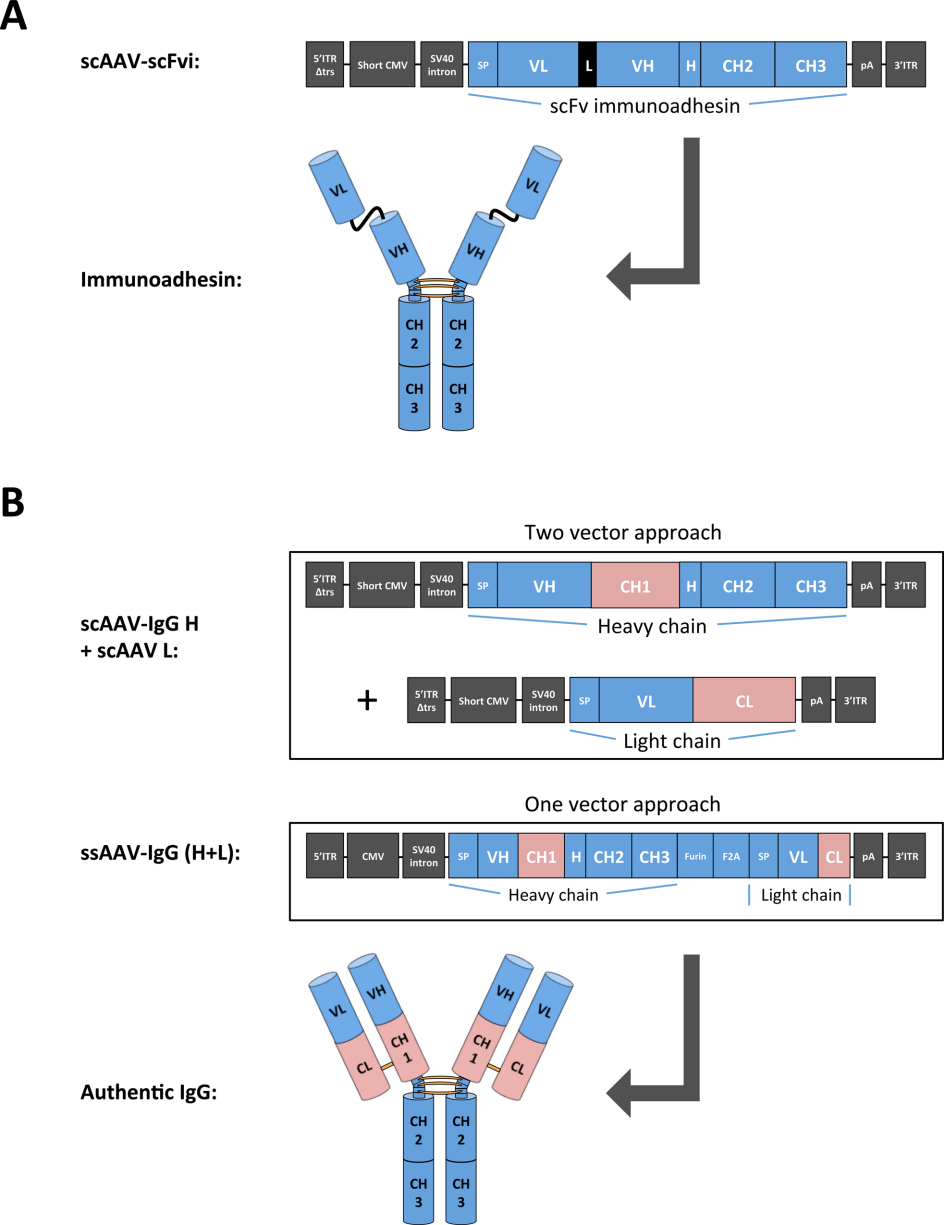
Schematic illustration of AAV constructs and transgene products. Design of recombinant AAV vectors expressing antibody or antibody-like molecules. (**A**) Self-complementary AAV (scAAV) containing an expression cassette for a single-chain fragment variable immunoadhesin (scFvi). Upon expression, the scFvi dimerizes to form a mature immunoadhesin with a MW of approximately 120 kDa. The expression cassette is flanked by AAV2 inverted terminal repeats (ITRs); the 5' ITR is truncated to form double-stranded AAV genomes [43,52]. (**B**) Two strategies for achieving expression of full-length antibodies. The first approach, called the two vector approach, requires two scAAV vectors, one encoding IgG heavy chain and one the light chain. The second strategy, called the one vector approach, utilizes one single-stranded AAV (ssAAV) vector only, with heavy and light chains of IgG expressed from one open reading frame. The two polypeptide chains are separated by a 2A peptide from foot-and-mouth-disease virus (F2A) that mediates cleavage and a furin peptide that allows removal of redundant amino acids at the heavy chain C-terminus following furin enzyme-dependent cleavage. Thus, the heavy chain C-terminus is believed to attain an authentic sequence. The light chain N-terminus is believed to gain an authentic sequence following signal peptide (SP)-mediated cleavage. The transgene cassette is flanked by AAV2 ITRs to form single-stranded AAV genomes. The full-length authentic IgG has a MW of ≥ 150 kDa. Abbreviations: 5'ITRΔtrs, 5' inverted terminal repeat devoid of the terminal resolution site; Short CMV, a shortened variant of the immediate early CMV promoter (CMV) [16]; SV40 intron, an intron from simian virus 40; SP, signal peptide; VL, variable light domain; L, serine-glycine linker peptide; VH, variable heavy domain; H, hinge region; CH, constant heavy domain; CL, constant light domain; pA, polyadenylation signal; Furin, cleavage sequence for the cellular protease furin.

Generation of full-length IgG requires AAV vector design to integrate the coding sequences of both heavy and light chains. Early studies demonstrated the feasibility of expressing a monoclonal antibody (mAb) from a single rAAV vector by employing a dual promoter approach, albeit with modest efficiency [26]. Similarly, incorporation of internal ribosomal entry sites (IRES) into transgene expression cassettes was shown to have limited efficiency with regard to equimolar expression levels [27]. Development of bicistronic vectors that use only one promoter and a 2A self-processing sequence from foot-and-mouth disease virus (F2A) have created improved antibody expression systems [28]. Modified, double-stranded rAAV vectors, termed scAAVs, were reported to achieve higher transduction efficiencies than conventional ssAAVs but are limited in their packaging capacity that is only half that of ssAAV [22]. To explore the performance of both the scAAV and ssAAV vector systems side-by-side, we designed rAAVs encoding the heavy and light chains of the anti-SIV antibodies 4L6 and 5L7 as illustrated in (Fig 1). These Ab constructs were compared to their shorter immunoadhesin counterparts [16].

**Fig 2:**
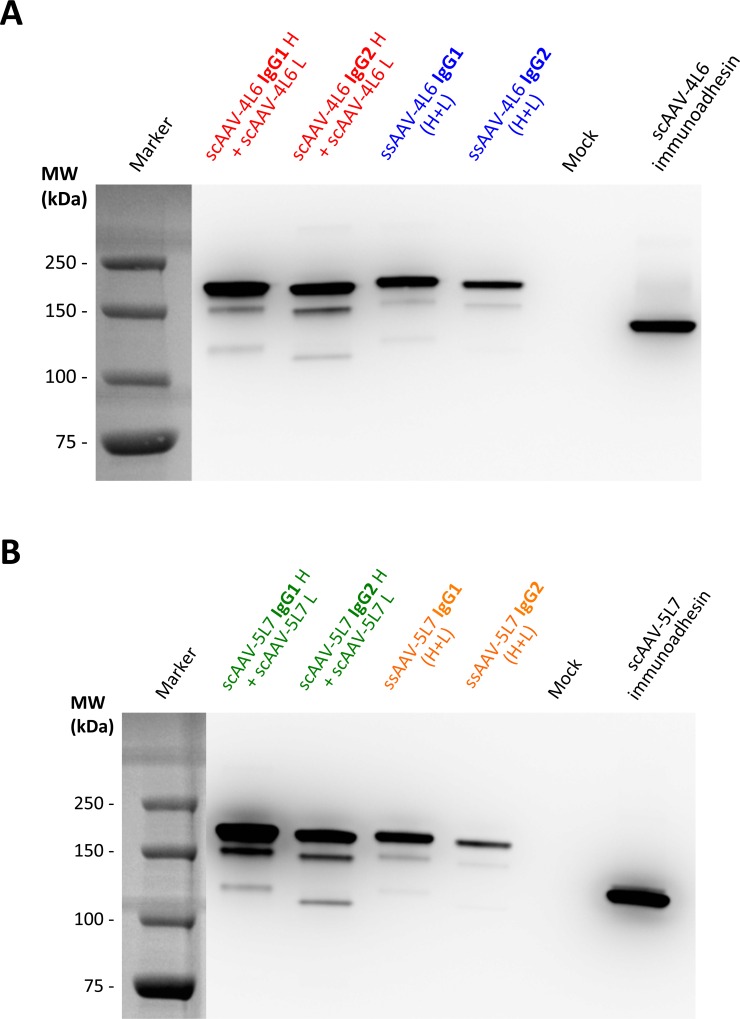
Expression of full-length antibodies from recombinant AAV vectors. Levels of expressed IgG or immunoadhesin were analyzed by Western Blot after transfection of HEK293T cells with equal amounts of plasmid DNA (0.5 μg + 0.5 μg or 1 μg). Comparison of secreted (**A**) 4L6 IgGs or (**B**) 5L7 IgGs from co-transfection of heavy and light chain vectors (two vector approach) *vs*. transfection of bicistronic vectors (one vector approach). The two vector approach yielded slightly higher levels of secreted antibodies than the one vector approach. IgG1 versions of the 4L6 and 5L7 full-length antibodies expressed better than IgG2 versions.

Coding sequences of the utilized Abs and immunoadhesins were codon-optimized using the OptimumGene algorithm for enhanced expression, gene-synthesized and cloned into AAV vector plasmids. HEK293T cells were then transfected with ssAAV vector plasmids that encode both the heavy and light chains of 4L6 IgG. Single vector transfection was compared to co-transfection of scAAV vector plasmids encoding 4L6 IgG heavy chain and 4L6 light chain; both sets used either the heavy chain of rhesus IgG1 or the heavy chain of rhesus IgG2. Both approaches yielded measurable levels of 4L6 IgG in the cell culture supernatant as determined by Western blot analysis (Fig 2A). Comparable expression was achieved following transfection with a single vector plasmid *vs*. transfection with two vector plasmids; similarly, full-length IgGs showed similar expression levels as the immunoadhesin derivative. Since transfections were normalized on a per μg basis of total plasmid DNA, levels of expressed IgG were likely to have been somewhat higher in these experiments with the two vector approach on a molar basis. Expression levels of 4L6 IgG2 appeared to be consistently lower than 4L6 IgG1 using both approaches. Similar results were obtained for the 5L7 IgGs (Fig 2B).

### Improving expression of IgG from ssAAV

**Fig 3:**
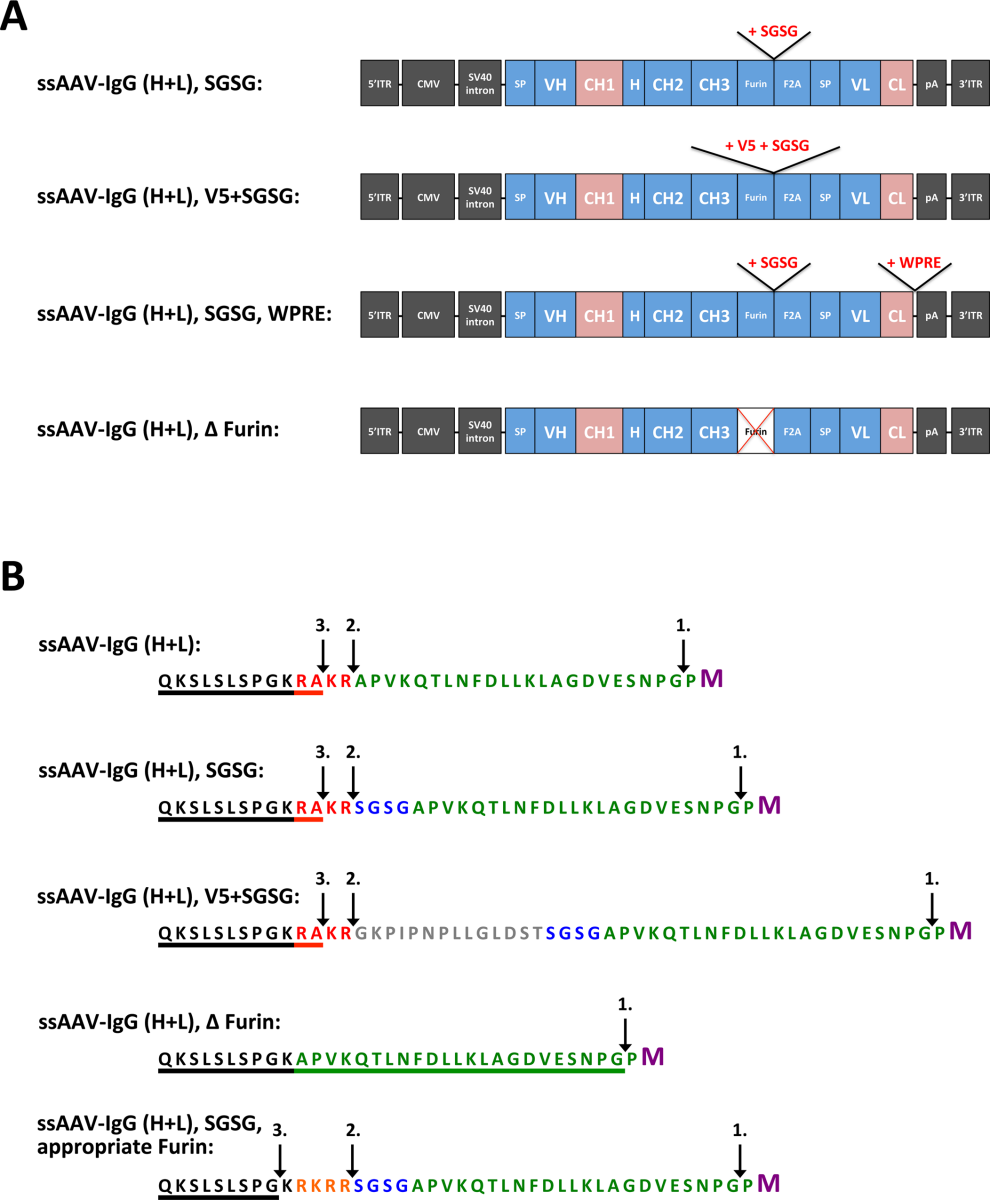
Variations to ssAAV vector design. Schematic illustration of modifications to the basic bicistronic ssAAV vector. (**A**) Variants of ssAAV vector comprise constructs with modifications to the Furin/F2A cleavage site and the 3' untranslated region (UTR); the changes include the addition of SGSG peptide and V5 peptide, deletion of furin peptide, and inclusion of a post-transcriptional regulatory element from woodchuck hepatitis virus (WPRE). Abbreviations: ITR, inverted terminal repeat of AAV2; 5'ITRΔtrs, 5' inverted terminal repeat devoid of the terminal resolution site; Short CMV, a shortened variant of the immediate early CMV promoter; SV40 intron, an intron from simian virus 40; SP, signal peptide; VL, variable light domain; L, serine-glycine linker peptide; VH, variable heavy domain; H, hinge region; CH, constant heavy domain; CL, constant light domain; pA, polyadenylation signal; Furin, cleavage sequence for the cellular protease furin; F2A, 2A peptide from foot-and-mouth-disease virus. (**B**) Amino acid sequences of the Furin/F2A cleavage site in the different ssAAV vector constructs. Amino acids were colored to illustrate the range of specific sequences: encoded sequence of the heavy chain C-terminus (black), Furin peptide (red/orange), V5 peptide (gray), SGSG peptide (blue), F2A peptide (green) and the first amino acid (M, Methionine) of the light chain signal peptide (purple). Cleavage sites are indicated by arrows and are numbered in the order that cleavage is believed to occur: F2A self-cleavage (1.), Furin enzyme-mediated removal (2.), carboxypeptidase enzyme-mediated cleavage of basic amino acids (3.). The underlined amino acids represent the heavy chain C-terminus after the final cleavage within the secretory pathway. In B-cells, the heavy chain genes encode the amino acids Pro-Gly-Lys (PGK) at the C-terminus, however, the secreted IgG lacks the terminal Lys (K) due to removal by carboxypeptidases [31]. The ssAAV-4L6 IgG1 vector construct containing the appropriate Furin peptide was utilized in Fuchs et al. [17].

**Fig 4:**
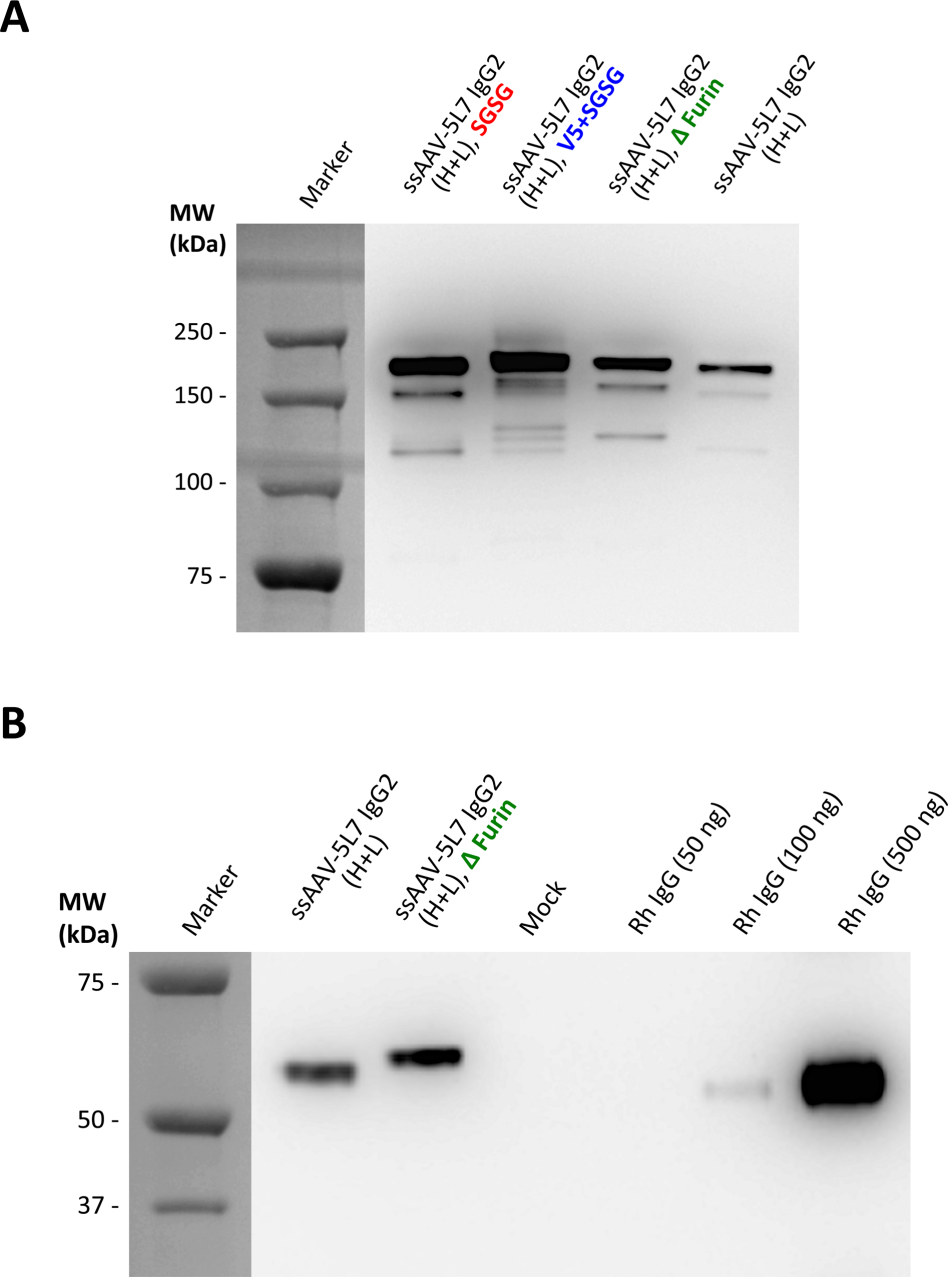
Levels of IgG expression from modified ssAAV vectors. Levels of expressed 5L7 IgG2 were analyzed by Western Blot after transfection of HEK 293T cells with equal amounts of bicistronic ssAAV vectors (1 μg). (**A**) The conventional ssAAV-5L7 IgG2 vector (as illustrated in Fig 1B) was modified by addition or deletion of peptides (SGSG, V5, Furin) and compared to each other. While all modifications improve expression of the antibody, only the SGSG version of the vector mediates correct F2A-Furin cleavage comparable to the conventional bicistronic ssAAV vector. (**B**) Demonstration of Furin-mediated cleavage of the F2A peptide remaining on the IgG heavy chain. Deletion of Furin peptide prevents removal of redundant amino acids from the heavy chain C-terminus following F2A cleavage.

We next examined the effects of a number of modifications to the ssAAV vector design. Our basic, bicistronic, ssAAV vector design included a peptide cleavage sequence for the cellular protease furin and a F2A self-cleaving peptide sequence between the heavy and light chains as previously described [28,29] (Fig 1B). Inclusion of the four amino acid linker peptide SGSG, or a V5 peptide sequence plus SGSG, following the furin peptide sequence and prior to the F2A peptide sequence was previously reported to increase expression of T-cell receptor (α and β chains) from lentiviral vectors [30]. We thus tested the effects of these modifications on expression of 5L7 IgG2 from our ssAAV bicistronic vector (Fig 3). Both modifications (SGSG alone or V5 plus SGSG) yielded measurably higher levels of 5L7 IgG2 production than when these peptide linkers were omitted (Fig 4A). Deletion of the Furin peptide from the original expression cassette resulted in somewhat higher expression of 5L7 IgG2 with the drawback of a shift in size of the heavy chain (Fig 4B). This result indicates, as expected, that amino acids derived from F2A cleavage were still present at the C-terminus of the heavy chain (Fig 3B). Although the V5 peptide afforded the 5L7 IgG2 to be favorably expressed, addition of the peptide generated heterogeneous transgene products as seen on the full-length IgG (Fig 4A). Please note that inclusion of an appropriate furin cleavage peptide sequence (RKRR) allows production of a heavy chain with a C-terminal sequence identical to the authentic molecule (Fig 3B) [29,31].

**Fig 5:**
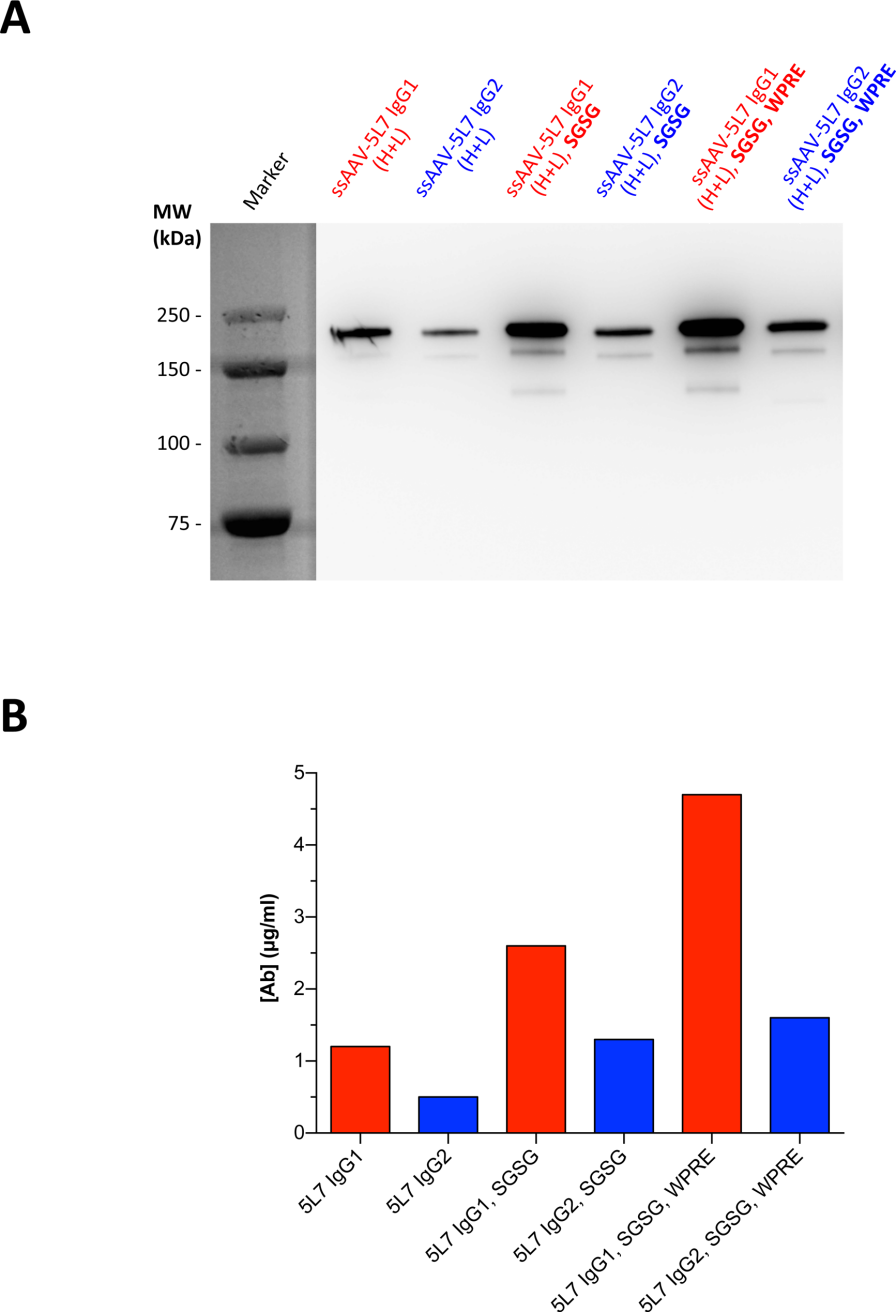
Further improvements of ssAAV vector expression cassettes. The exact same samples were analyzed by two methods. Yields of secreted 5L7 IgG antibodies were compared by (**A**) Western Blot and quantified by (**B**) ELISA, following transfection of HEK293T cells with different ssAAV vector plasmids.

We next incorporated a *cis*-acting element, termed WPRE from the woodchuck hepatitis virus (WHV), into our vector design in order to test its ability to increase expression of IgG from our ssAAV constructs (Fig 3A). This viral post-transcriptional regulatory element (PRE) elevated transgene expression in previous studies [32,33]. Incorporation of WPRE into our IgG expression cassettes noticeably increased the yield of measurable 5L7 IgG in cell culture supernatant (Fig 5A). The amount of secreted 5L7 IgG1 was 4-fold higher when expressed from the construct containing SGSG and WPRE compared to expression from the unmodified construct as determined by ELISA (Fig 5B). Modifications to the ssAAV vector plasmids expressing 5L7 IgG2 resulted in more than 2-fold higher protein yields (Fig 5B).

**Fig 6:**
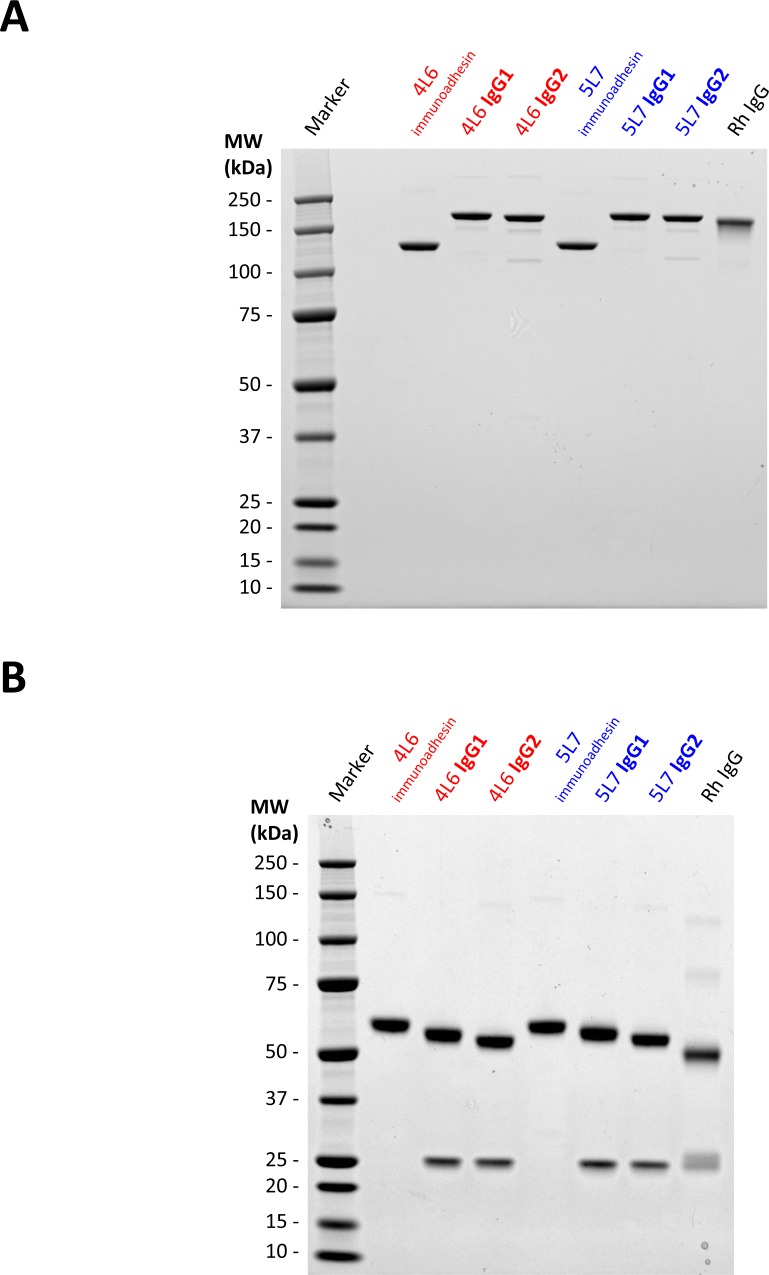
Coomassie staining of purified antibodies and immunoadhesins. Purity and integrity of purified IgGs and immunoadhesins was verified by coomassie staining following large-scale transfection of HEK293T cells with ssAAV-IgG and scAAV-immunoadhesin vector plasmids. SDS-PAGE (1 μg of purified protein per lane) and staining under (**A**) non-reducing and (**B**) reducing conditions. Both conditions confirmed the expected size and composition of the tested proteins. The immunoadhesins and full-length IgG versions of 4L6 and 5L7 have unusually long heavy chain CDR3 regions compared to polyclonal rhesus IgG heavy chains, thus, heavy chains of 4L6 and 5L7 have a considerably higher MW.

As a measure of quality control, we performed large-scale transfection of 293T cells using the optimized AAV vector plasmids and analyzed the purified IgGs. The purified proteins were separated by polyacrylamide gel electrophoresis under non-reducing (Fig 6A) and reducing conditions (Fig 6B). Full-length IgGs of 4L6 and 5L7 displayed expected band sizes when compared to purified total rhesus IgG and purified immunoadhesins. The observed higher molecular weights of the 4L6 and 5L7 heavy chains are likely a result of the unusually long CDR3 (complementarity determining regions) sequences present in these Abs [17,21]. Little or no aggregation or degradation products were observed and purified proteins were found to be of high purity as determined by Coomassie staining (Fig 6).

### *In vitro* transduction using rAAV

**Fig 7:**
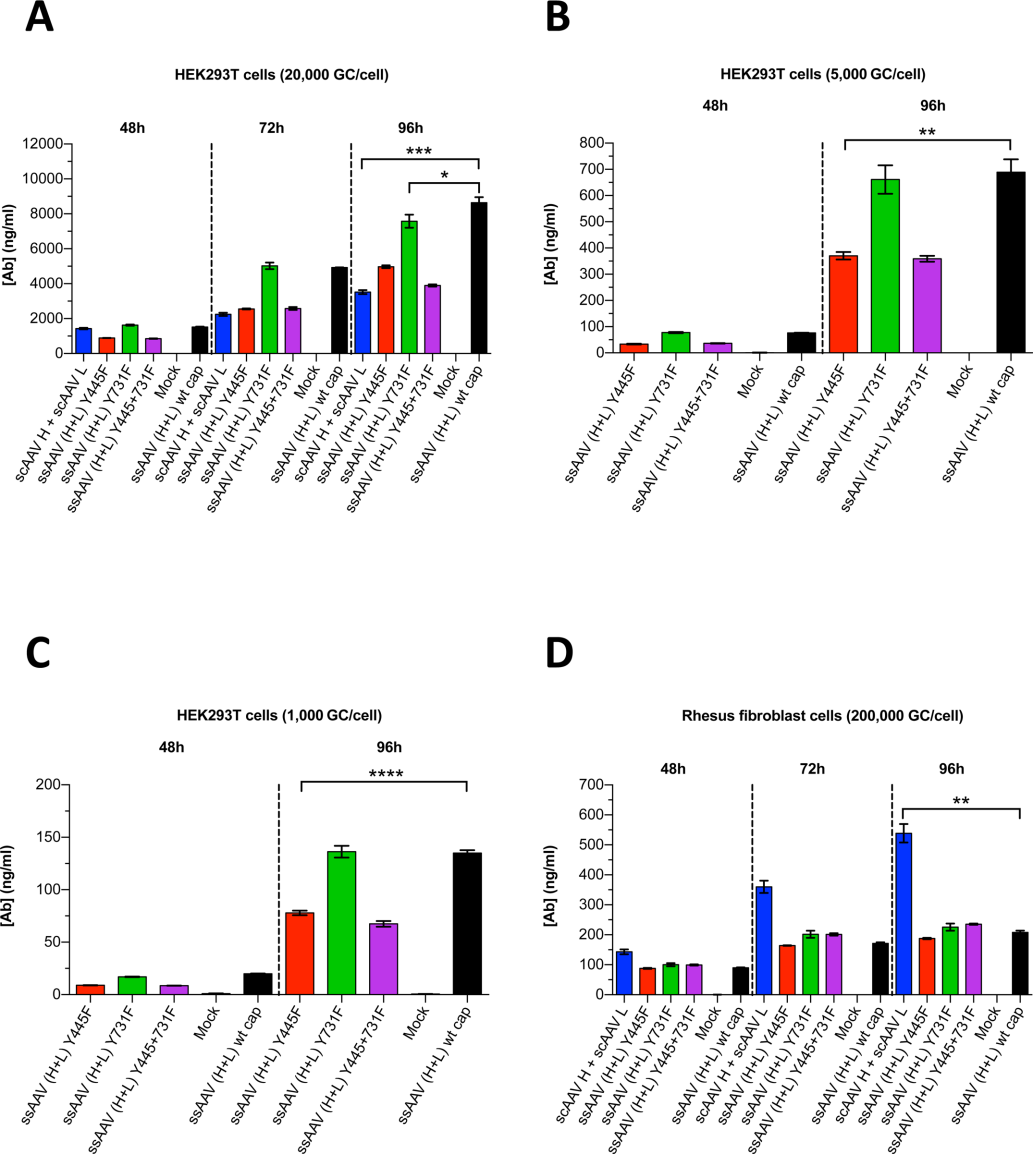
Expression of 5L7 IgG1 after AAV-mediated transduction *in vitro*. AAV vectors were encapsidated with AAV1 wild-type (wt) capsid or AAV1 mutant capsids (Y445F and/or Y731F); in the case of ssAAV, we utilized the modified ssAAV vector construct containing both SGSG and WPRE. Purified AAV virus particles were then used for transduction. HEK293T cells were infected with (**A**) 2x10^4^ rAAV genome copies per cell (GC/cell), (**B**) 5x10^3^ GC/cell and (**C**) 1x10^3^ GC/cell. (**D**) Rhesus fibroblast cells were infected with 2x10^5^ GC/cell. AAV transduction experiments shown in (**A** + **D**) were conducted at a different time than experiments in (**B** + **C**). Levels of secreted antibody were measured by ELISA following the time of transduction. Values are depicted as mean ± SD (n = 3/group); ****p < 0.0001, ***p < 0.001, **p < 0.01, *p < 0.05.

Several groups have studied the influence of AAV capsid mutations on the transduction efficiency of AAV vectors. Notably, capsid mutants Y444F and Y730F for AAV serotype 2 and Y445F and Y731F for AAV serotype 6 were reported to have markedly improved transduction efficiencies *in vitro* and *in vivo* [34–37]. The amino acids Y445 and Y731 in AAV6 are correspondingly located in AAV1. We thus investigated the effects of these mutations in the context of our AAV1 vectors on transduction/expression efficiency. Recombinant AAV vector plasmids containing SGSG and WPRE were used for large-scale transfection of 293T cells to generate rAAV1 particles; the method included a helper plasmid containing adenoviral genes and another plasmid containing the genes *rep* from AAV2 and *cap* from AAV1. While in one set of transfections we employed AAV1 wild-type *cap*, other sets included the AAV1 capsid mutants Y445F and/or Y731F. HEK293T cells and rhesus fibroblast cells were then infected with normalized doses of rAAVs encoding the 5L7 IgG1 antibody. Cell culture supernatants were harvested 48 to 96 h after transduction and the amount of secreted 5L7 IgG1 was measured by ELISA. The concentration of 5L7 IgG1 increased with time following each collection of supernatant and the levels were dependent on the inoculum dose (MOI) (Fig 7). Using the AAV1 capsid mutants Y445F and Y731F in the context of ssAAV achieved no measurable enhancement on the yield of secreted Ab in either cell type (Fig 7A–7C and 7D). Co-transduction of heavy and light chain vectors in scAAV yielded significantly higher levels of 5L7 IgG1 than transduction using ssAAV in rhesus fibroblast cells (Fig 7D). In HEK293T cells, the opposite was true (Fig 7A–7C). The differences in the observed levels of 5L7 IgG1 secretion between the scAAV and ssAAV vectors that were used were not large but they were statistically significant (Fig 7).

## Discussion

Considering its record of safety [9,11,13,14,38,39] and its potential for long-term efficacy [10,13,14,39–41], rAAV will likely be increasingly utilized against diseases for which conventional treatments do not exist, have not succeeded, or are insufficient. Development of an effective vaccine against HIV/AIDS and long-term virological control of HIV in the absence of daily drugs have proven to be elusive goals for the field. These are areas where use of rAAV to achieve long-term delivery of potent broadly-neutralizing anti-HIV mAbs could make a significant impact. AAV-mediated delivery of anti-HIV/SIV antibodies or antibody-like molecules has already demonstrated effectiveness in mice [20] and monkeys [16–19]. Protective efficacy has been largely dependent on serum concentration, persistence, and potency of the AAV-delivered antibodies.

Plasma levels of any AAV-delivered antibody will depend on the efficiency of expression, the half-life of the antibody, and the extent to which there are immune responses to the transgene product. Factors that influence the efficiency of AAV-mediated expression of antibodies include: AAV serotype, vector dose, route of administration, transduction efficiency of the rAAV, amino acid sequence of the transgene product, codon usage of the transgene, and the composition of the vector design. High transduction efficiencies have been reported with the use of scAAV vectors [16,23,42]. However, scAAV vectors cannot package the entire coding sequence of an authentic IgG; consequently, the IgG heavy and light chains need to be provided by two separate vectors, which can lead to unequal pairing of the two polypeptide chains. Previous studies demonstrated that F2A could be used to express H and L chains in approximately equimolar amounts [28] and that the entire coding sequence of IgG could be packaged by a single ssAAV [17,19,20,28]. Here we confirmed the applicability of using F2A in ssAAV by expressing the anti-SIV antibodies 4L6 and 5L7. In the context of AAV-mediated transduction in cell culture, co-administration of heavy and light chain vectors with scAAV (two vector approach) yielded levels of secreted antibody that were only somewhat higher than those that were achieved with our improved bicistronic vector constructs (one vector approach) in early passage rhesus fibroblasts. However, while the two vector approach performed better than the one vector approach in rhesus fibroblast cells, it showed no enhanced performance for the production of secreted 5L7 IgG1 in HEK293T cells. Considering the extensive literature on the enhanced performance of scAAV vectors [22,23,42–45], these results may seem surprising. However, it is important to note that in our case two polypeptides (H and L) must come together appropriately for the IgG to be formed and secreted. It is also important to note that the ssAAV that was used had modifications to enhance performance. These as well as other factors could have affected the relative performance of scAAV *vs*. ssAAV in our experiments.

We were able to increase the yield of expressed Ab by introducing modifications to the ssAAV vector design. Our optimization method followed a study conducted by Yang et al. [30] that demonstrated improved T-cell receptor gene expression by extending the F2A cleavage site. Inclusion of the SGSG peptide between the Furin peptide and F2A had a similar enhancing effect in our experiments. The addition of V5 peptide increased the expression of our 5L7 Ab even more but it appeared to produce heterogeneous Ab products, which could be a result of insufficient proteolytic cleavage due to a high demand of the cellular Furin enzyme or steric hindrance caused by the V5 peptide itself. Using an error-prone expression cassette could be detrimental *in vivo* if the V5 peptide triggered anti-V5 immune responses. We sought to increase IgG expression even further by including WPRE into the ssAAV expression and we confirmed higher yields of measurable Ab. Inclusion of the SGSG peptide and WPRE elevated expression of our tested Abs up to 4-fold compared to non-modified ssAAV vectors. 5L7 IgG delivered to monkeys by intramuscular inoculation of AAV1 vector using our single-stranded (SGSG and WPRE) and self-complementary vector designs was previously shown to bind SIV envelope glycoprotein 140 (gp140) and to retain full SIV neutralizing activity [17]; thus, 5L7 IgG produced by AAV from these vector designs is fully functional. Use of WPRE in clinical trials, however, should be considered with care as certain WPRE sequences might have potential oncogenic activity [46]; in contrast, modified PREs have been reported to represent a safe alternative [47].

AAV-mediated transduction requires high numbers of rAAV particles to be injected *in vivo*. If the administered AAV vector dose is too high, anti-AAV capsid immune responses can negatively influence the outcome of AAV-mediated gene transfer leading to a loss of efficacy [10,41,48,49]. Previous studies explored the possibility of increasing the efficiency of rAAV vectors by improving their ability to transduce cells; success along these lines may allow a lower vector dose and avoid unwanted immune responses to AAV capsid. Here we compared for the first time ssAAV vector with AAV1 wild-type capsid to ssAAV vectors with AAV1 capsid mutations Y445F and Y731F for their ability to transduce cells in culture. In contrast to previous studies that tested these mutations in the context of AAV2 [34,35] and AAV6 [37] capsid, we observed no enhancing effect for AAV1 capsid mutations Y445F and Y731F with regard to transduction efficiency. Previous results with AAV1 capsid mutants Y445F and Y731F in dogs were not compared to the AAV1 wild-type capsid [36]; therefore, possible enhancing effects could not be estimated from that study. Nonetheless, mutations of other amino acids in the AAV1 capsid or use of other cell types could still potentially increase transduction efficiency as observed with AAV2 and AAV6. Also, animal experiments will need to be performed in order to evaluate the efficiency of IgG delivery using AAV1 with Y445F and Y731F substitutions.

In summary, we have studied the effects of stepwise changes to vector design with the goal of improving antibody production from rAAV vectors. Several modifications to ssAAV vector were found to increase IgG expression; our observations will hopefully facilitate decision making regarding vector design for future use of AAV for antibody delivery.

## Material and Methods

### Plasmid DNA construction

Coding sequences of the 4L6 and 5L7 antibodies (heavy chain, light chain or bicistronic) were designed *in silico* and codon-optimized using the proprietary OptimumGene algorithm (Genscript), which takes into consideration multiple critical factors involved in different stages of gene expression, e.g., codon usage bias, GC content, mRNA secondary structure, RNA instability motifs and repeat sequences. Subsequently, the optimized coding sequences were gene-synthesized (Genscript). The bicistronic expression cassettes further contained F2A peptide [28] and a Furin peptide [29]; additional peptides (V5 and SGSG) were included as indicated [30]. 4L6 and 5L7 immunoadhesin sequences [16,17] served as a template and full-length antibodies were constructed by adding CH1 domain and CL domain of rhesus IgG to the already known immunoadhesin sequences. 4L6 and 5L7 sequences originate from recombinant anti-SIV Fab sequences derived from the bone marrow of SIV-infected rhesus monkeys [50]. Rhesus IgG1 sequence is based on accession no. AAF14058 and AAQ57555, and rhesus IgG2 sequence is based on AAF14060 and AAQ57567. Rhesus kappa light chain was designed using CL domain sequence from AAD02577. Synthesized fragments were then cloned into NotI site of scAAV or ssAAV vector plasmids [16]. Where indicated, ssAAV was modified by insertion of WPRE, a post-transcriptional regulatory element (PRE) of woodchuck hepatitis virus (WHV). WPRE sequence is based on nucleotides 1093–1684 of the WHV genome (accession no. J04514) [32]. The WPRE DNA fragment was gene-synthesized (Genscript) and cloned into NotI site of the ssAAV vector plasmid, downstream of the transgene Stop codon and prior to the polyA site of the expression cassette.

### Cell culture and DNA transfection

HEK293T/17 cells (ATCC) were maintained in complete D10 growth medium: DMEM supplemented with 10% ultra-low IgG FBS, 25 mM HEPES, 2 mM L-glutamine (all Gibco, Thermo Fisher) and 100 μg/ml Primocin (InvivoGen). Rhesus macaque skin fibroblasts (NEPRC, Harvard Medical School) were maintained in complete D20 growth medium: DMEM supplemented with 20% ultra-low IgG FBS, 25 mM HEPES, 2 mM L-glutamine (all Gibco, Thermo Fisher) and 100 μg/ml Primocin (InvivoGen). Cells intended for transfection were seeded in complete growth medium into 6-well or 12-well CellBIND plates (Corning) 1 day prior to transfection. On the day of transfection, cells reached a confluency of 50 to 70% and were transfected with the recommended amount of DNA using jetPRIME buffer and jetPRIME reagent (both Polyplus-transfection). Cell culture medium was changed 12 to 24 h after transfection by removing the complete growth medium and replacing it with half the volume of serum-free medium (medium that lacked FBS and Primocin). Cell culture medium was harvested 72 to 96 h after transfection and clarified supernatant was obtained by centrifugation at 16,000 RCF and 4°C for 15 min.

### Immunoblotting

Cell culture supernatant was tested for secreted antibodies by Western blot. Proteins were separated by non-reducing SDS-PAGE on 4 to 12% Bis-Tris gels (NuPAGE, Thermo Fisher) and transferred onto PVDF membranes by semi-dry blotting (Trans-Blot SD, Bio-Rad). The membranes were blocked with 1x PBS (Gibco, Thermo Fisher) containing 5% nonfat dry milk (Bio-Rad) for 30 min to 1 h at room temperature. Afterwards, a HRP-conjugated goat anti-rhesus IgG (H+L) antibody (SouthernBiotech) was applied (1:2000 to 1:5000 in blocking buffer) and membranes were incubated for 1 to 2 h at room temperature or overnight at 4°C. The membranes were then washed 5 times using 1x PBS containing 0.05% TWEEN 20 (Sigma-Aldrich) and chemiluminescence was detected by using the SuperSignal West Pico or Femto chemiluminescent substrate (Pierce, Thermo Fisher) and a LAS3000 chemiluminescence imager (FujiFilm).

### Antibody quantification by ELISA

Concentration of secreted antibody in cell culture supernatant was measured by ELISA. Test plates were coated with 5 μg/ml (diluted in 1x PBS) purified unlabeled Protein A of *Staphylococcus aureus* (SouthernBiotech) for 1 h at 37°C. Plates were washed with 1x PBS containing 0.05% TWEEN 20 (Sigma-Aldrich) and then blocked with 5% nonfat dry milk (Bio-Rad) in 1x PBS for 1 h at 37°C. Purified IgG of *Macaca mulatta* (Immune Technology) as standard and cell culture supernatants were serially diluted 1:3 in blocking buffer and added to the test plates. After 1 h of incubation at 37°C the plates were washed again and a diluted (1:5000 in blocking buffer) HRP-conjugated goat anti-rhesus IgG (H+L) (SouthernBiotech) was added. The reaction was stopped after 1 h at 37°C and plates were washed 10 times. Subsequently, TMB substrate and stop solution (SouthernBiotech) were added and absorbance at 450 nm was measured in a microplate reader (PerkinElmer).

### Production, purification and analysis of antibodies

HEK293T/17 cells (ATCC) were seeded in complete growth medium into T225 flasks (Corning) 1 day prior to transfection. On the day of transfection, cells reached a confluency of 50 to 70% and were transfected with the recommended amount of DNA using jetPRIME buffer and jetPRIME reagent (both Polyplus-transfection). Cell culture medium was changed 12 to 24 h after transfection by removing the complete growth medium from each flask and replacing it with 60 ml of serum-free medium. Cell culture medium was harvested 72 to 96 h after transfection and clarified supernatant was obtained by centrifugation at 4,000 RCF and 4°C for 15 min, and subsequent filtration through a 0.2 μm PES rapid-flow filter (Nalgene, Thermo Scientific). Full-length antibodies and immunoadhesins were purified over Protein A Plus agarose (Pierce, Thermo Fisher) following the instructions of the manufacturer. Purified proteins were separated by reducing and non-reducing SDS-PAGE on 4 to 12% Bis-Tris gels (NuPAGE, Thermo Fisher). The gels were then stained with Coomassie G-250 following the SimplyBlue SafeStain protocol (Invitrogen, Thermo Fisher).

### Recombinant AAV and *in vitro* transduction

Production of recombinant AAV (rAAV) was conducted as described previously [51]. In short, HEK293 cells (ATCC) were transfected with rAAV vector plasmid and two helper plasmids to allow generation of infectious AAV particles. The helper plasmid that contained the AAV cap gene had either a wild-type or mutant genotypes: AAV1 wild-type (wt) capsids and AAV1 mutant capsids (Y445F and Y731F). After harvesting transfected cells and cell culture supernatant, rAAV was purified by three sequential CsCl centrifugation steps. The vector genome copy number (GC/ml) was assessed by Real-Time PCR (S1 Table). The integrity of AAV particles was verified by electron microscopy (EM) (S1 Fig) and the purity of the AAV preparations was verified by silver-stained SDS-PAGE (S2 Fig). Cells intended for AAV transduction were seeded in complete growth medium into 6-well or 12-well CellBIND plates (Corning) 1 day prior to transduction. On the day of AAV transduction, cells reached a confluency of 50 to 70% and were infected with a total of 2x10^4^ (HEK 293T) and 2x10^5^ (rhesus fibroblast) rAAV particles per cell. In cases where two scAAV vectors were used, equal amounts of heavy and light chain vectors were mixed to yield the total amount of AAV. Cell culture medium was changed 12 to 24 h after transduction by removing the complete growth medium and replacing it with half the volume of serum-free medium. Cell culture medium was harvested from separate plates at 48 h, 72 h and 96 h after transduction, and clarified supernatant was obtained by centrifugation at 16,000 RCF and 4°C for 15 min. Concentration of secreted 5L7 IgG1 in cell culture supernatant was measured by Protein A/anti-rhesus IgG ELISA using purified rhesus IgG as standard as described above.

### Statistical analysis

Data analysis was performed using Prism (GraphPad Software). Groups were compared by two-tailed, unpaired *t* test with Welch correction. All values were depicted as mean ± standard deviation (SD). A *p* value of < 0.05 was considered significant.

## Supporting Information

S1 Fig Electron microscopy (EM) images of recombinant AAV particles used for AAV-mediated transduction *in vitro*. Each AAV preparation was scrutinized by EM to verify morphology and ultrastructure of produced recombinant AAV particles. Purified AAV particles were spread on a freshly prepared carbon-coated Formvar support film and stained with 1% uranyl acetate. The large field of virus particles was visualized with a transmission electron microscope (TEM) at 92,000x magnification. EM analysis was done by the EM core of the University of Massachusetts. The bar on the lower left side represents 200 nm.
(TIF)

S2 Fig Silver-stained SDS-PAGE of purified AAV particles. In each AAV preparation the three AAV capsid proteins VP1, VP2 and VP3 were visualized by silver staining on a polyacrylamide gel. The AAV particles are composed of AAV1 wild-type (wt) capsid or AAV1 mutant capsids (Y445F and/or Y731F).
(TIF)

S1 Table Vector genome titer of recombinant AAV stocks (GC/ml) as determined by Real-Time PCR. (TIF)
