# Supplementary figures and images for: Recombinant AAV Vectors for Enhanced Expression of Authentic IgG
Source: PLoS One. 2016 Jun 22;11(6):e0158009. doi: 10.1371/journal.pone.0158009 (PMC4917256; doi:10.1371/journal.pone.0158009)

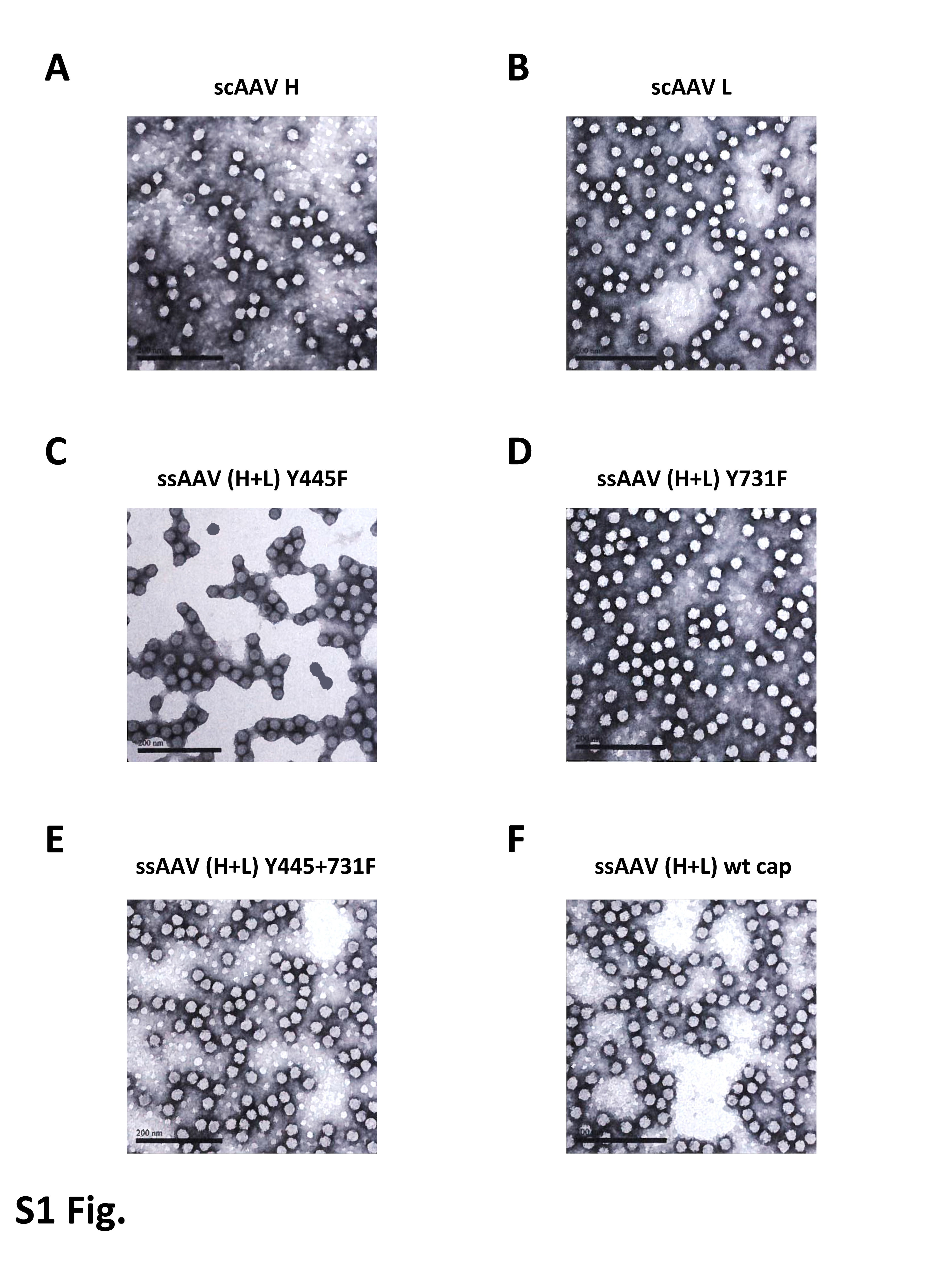

Supplement: S1 Fig — Each AAV preparation was scrutinized by EM to verify morphology and ultrastructure of produced recombinant AAV particles. Purified AAV particles were spread on a freshly prepared carbon-coated Formvar support film and stained with 1% uranyl acetate. The large field of virus particles was visualized with a transmission electron microscope (TEM) at 92,000x magnification. EM analysis was done by the EM core of the University of Massachusetts. The bar on the lower left side represents 200 nm. (TIF) [file pone.0158009.s001.tif]

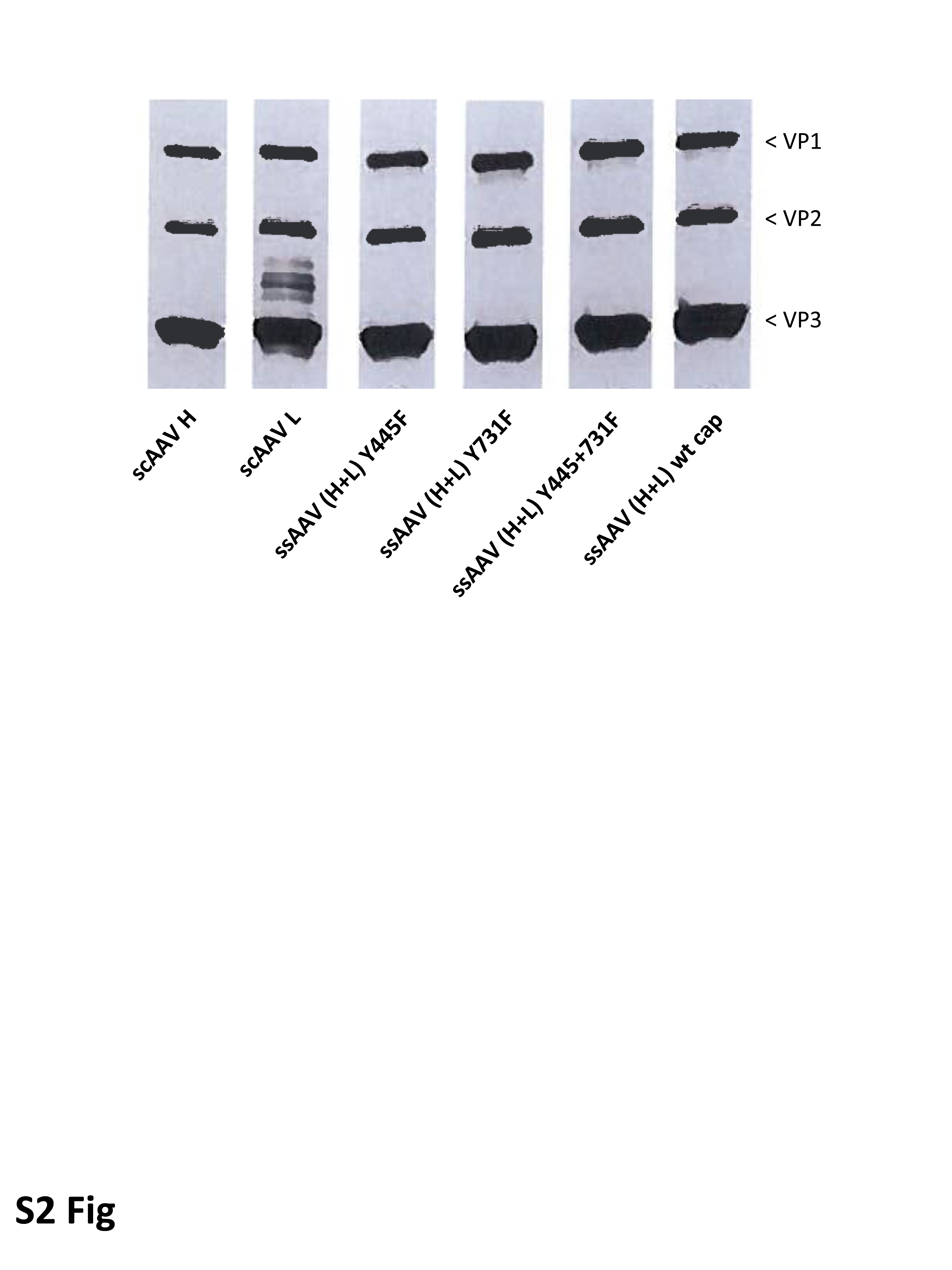

Supplement: S2 Fig — In each AAV preparation the three AAV capsid proteins VP1, VP2 and VP3 were visualized by silver staining on a polyacrylamide gel. The AAV particles are composed of AAV1 wild-type (wt) capsid or AAV1 mutant capsids (Y445F and/or Y731F). (TIF) [file pone.0158009.s002.tif]

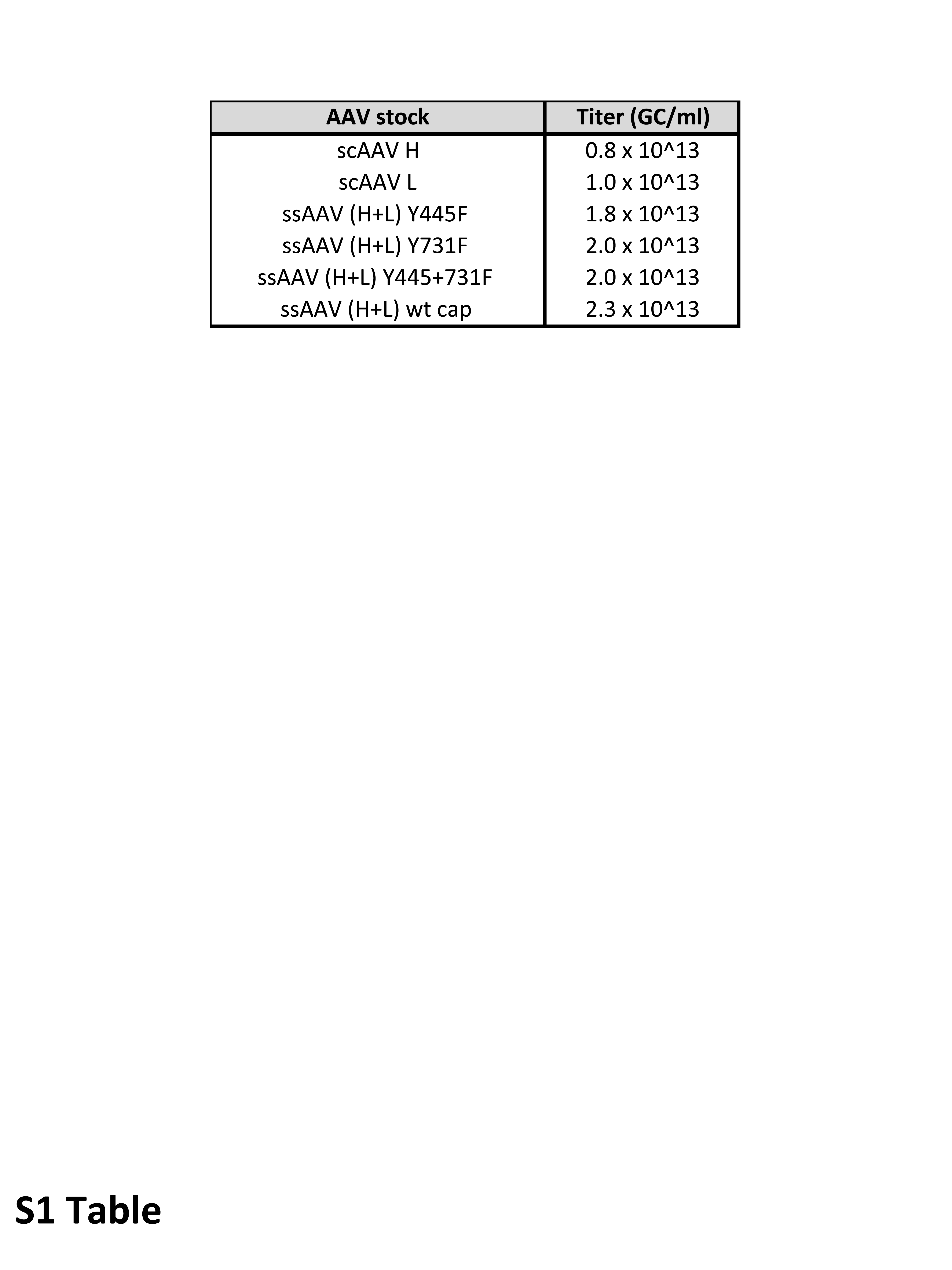

Supplement: S1 Table — (TIF) [file pone.0158009.s003.tif]
